# Enhancing the effectiveness of γδ T cells by mRNA transfection of chimeric antigen receptors or bispecific T cell engagers

**DOI:** 10.1016/j.omto.2023.08.003

**Published:** 2023-08-24

**Authors:** John Anderson, Marta Barisa

**Affiliations:** 1 UCL Great Ormond St Institute of Child Health, London, UK

The gamma delta (γδ) subset of human T cells is defined by the expression of a T cell receptor (TCR) comprised of a gamma and delta chain. The major subset in human peripheral blood expresses a Vγ9Vδ2 TCR and is sensitive to activation and expansion in the presence of aminobisphosphonate drugs such as zoledronic acid (ZA). Its ready availability as a clinical-grade reagent lends ZA for use in clinical applications to expand Vγ9Vδ2 cells to large numbers from peripheral blood—a GMP-compatible manufacturing backbone in which genetic modification can be evaluated.

In this issue of *Molecular Therapy - Oncolytics*, Becker and co-workers from the laboratory of Trent Spencer (https://doi.org/10.1016/j.omto.2023.05.007) have evaluated electroporation with mRNA of ZA-activated peripheral blood as an alternate strategy to viral transduction for genetic modification of Vγ9Vδ2 cells for anti-cancer therapeutic applications. Using the approach, the group successfully generated expanded γδ T cells that express a chimeric antigen receptor (CAR) or secreted bispecific T cell engagers (BiTEs). The resultant cell products enhanced γδ T cell cytolytic properties against antigen (CD19 or CD20)-positive B cell cancer targets compared with unmodified γδ T cells. While other groups have published approaches for expression of CARs in γδ T cells using conventional viral approaches,^1^^,^^2^ the development of electroporation protocols has some potential advantages that are of particular relevance to the utility of γδ T cells in adoptive immunotherapy.

Although Vγ9Vδ2 cells are the predominant subset of γδ T cells in human peripheral blood, non-Vγ9Vδ2 cells predominate in other organs (e.g., intestinal epithelial lymphocytes and skin-resident γδ T cells). Indeed, recent insights into the biological and oncological significance of these non-Vγ9Vδ2 γδ T cell subsets have contributed to an exponential increase in academic and commercial interest in developing γδ T cell products for cancer immunotherapy. Several published studies analyzing tumor-infiltrating γδ T cells functionally and phenotypically have pointed to an important role in tumor control. One of the earliest of such studies employed an unbiased approach for comparing tumor-infiltrating leukocyte roles across multiple cancer types. This study suggested a pivotal role for tumor-resident γδ T cells in immune surveillance and ranked γδ T cells as the most significantly associated with favorable prognosis of all subpopulations of infiltrating cells in human cancer, implying that their role is overall more for tumor control than as a regulatory subset.^3^ Of note, the majority of tumor-infiltrating γδ T subsets were of the non-Vγ9Vδ2 subset, a finding that has also been observed in neuroblastoma.^4^ More recently, Wu and colleagues investigated infiltrating Vδ1-TCR γδ T cells in healthy lung and lung cancer to identify and phenotype a tissue-resident population with effector function characteristics that might be of particular interest for evaluation in solid cancers.^5^ Recent studies point to Vδ1 and Vδ3 cells as being major players in immune responses to double checkpoint inhibition in cancers lacking major histocompatibility complex (MHC) class I expression.^6^ In this regard, it is encouraging that a number of groups have published protocols for expansion and a number of groups have published protocols for expansion and transduction with CAR-T of the Vδ1 subpopulation from blood and have obtained similar cell numbers in the therapeutic product as have been reported using the zoledronate protocols.^7^^,^^8^^,^^9^ Hence, the contribution of Becker et al. to increase the number of technologies available for gene modification of γδ T is timely and may have applications beyond ZA-expanded Vγ9Vδ2 cells.

We have cataloged the different approaches of γδ T cellular therapies for cancer that have been evaluated in the clinic over the last two decades, between the first trial in 2003 to the latest trial registered in 2022. We identified 77 γδ T cell intervention-based oncology clinical studies, with most studies being registered in Europe, China, and the USA. We predict that this will increase further, in particular in relation to the non-Vγ9Vδ2 subsets and gene-modified γδ T cell products.

Why is electroporation of mRNA for γδ T cell gene modification a clinically attractive approach? This can be addressed by considering the unique selling point of γδT in cancer adoptive immunotherapy, which is their potential for use as allogeneic off-the-shelf therapies. The MHC-unrestricted γδ-TCR repertoire, although potentially more diverse than that of αβ T cells by virtue of higher numbers of variable, joining, and diversity segments, lacks the capacity for self-reactivity seen with the αβ TCR. The implication of this is a lack of reactivity of allogeneic products with non-self MHC molecules and therefore a lack of graft versus host disease (GvHD). The first clinical trials demonstrating a lack of GvHD following infusion of genetically unmodified γδ T cells have thus far indicated a favorable safety profile, albeit with a lack of convincing clinical benefits.^10^

Hence, there is a research need to identify and clinically evaluate a range of genetic modifications to boost anti-cancer function and persistence of adoptively transferred allogeneic γδ T cells. In the proof-of-concept studies by Becker et al., single infusions of γδ T cells electroporated with both CAR and BiTe induce initial B cell cancer regressions, but of note, all treated animals show disease recurrence, indicating that the innate cytotoxic properties of the γδT are insufficient to maintain disease control after the synthetic receptor or secreted antibody properties naturally diminish. Disease control could be improved through repeat infusion, although this approach falls short of the ultimate clinical goal of a single engrafting and curative cell therapy.

Nevertheless, transient expression conferred by mRNA electroporation holds potential significant advantages in terms of facilitating translation. These specific advantages are (1) the relatively inexpensive generation of GMP RNA compared with the production costs of a single production run of GMP viral vector, (2) enhanced safety due to the short-lived nature of the genetic modification such that off-target or on-target off-tumor toxicities will be short lived, (3) additional safety benefits from the lack of integrating viral genomes, and (4) the emergence of electroporation as a clinically favored technique to facilitate GMP engineering of CRISPR base-edited T cell products. CRISPR base-edited αβ T cells are showing clinical traction,^11^ and long-term engraftment of allogeneic γδ T cells in the next generation of trials might require stealth strategies such as silencing of MHC to prevent graft rejection or insertion of chimeric receptors into endogenous promoters; for this, the utility of efficient electroporation and expansion protocols will be of value to the field.
